# Rapid eradication of vancomycin and methicillin-resistant *Staphylococcus aureus* by MDP1 antimicrobial peptide coated on photocrosslinkable chitosan hydrogel: *in vitro* antibacterial and *in silico* molecular docking studies

**DOI:** 10.3389/fbioe.2024.1385001

**Published:** 2024-04-11

**Authors:** Sarvenaz Ekhtiari-Sadegh, Saeed Samani, Farnoosh Barneh, Shirin Dashtbin, Mohammad Ali Shokrgozar, Kamran Pooshang Bagheri

**Affiliations:** ^1^ Venom and Biotherapeutics Molecules Lab, Medical Biotechnology Department, Biotechnology Research Center, Pasteur Institute of Iran, Tehran, Iran; ^2^ Department of Tissue Engineering, School of Advanced Technologies in Medicine, Tehran University of Medical Sciences, Tehran, Iran; ^3^ Department of Microbiology, School of Medicine, Iran University of Medical Sciences, Tehran, Iran; ^4^ National Cell Bank of Iran, Pasteur Institute of Iran, Tehran, Iran

**Keywords:** photocrosslinkable chitosan hydrogel, VRSA/MRSA, MDP1, antimicrobial peptide, eradication, molecular docking

## Abstract

**Introduction:**

Antibiotic resistance and weak bioavailability of antibiotics in the skin due to systemic administration leads to failure in eradication of vancomycin- and methicillin-resistant *Staphylococcus aureus* (VRSA and MRSA)-associated wound infections and subsequent septicemia and even death. Accordingly, this study aimed at designing a photocrosslinkable methacrylated chitosan (MECs) hydrogel coated by melittin-derived peptide 1 (MDP1) that integrated the antibacterial activity with the promising skin regenerative capacity of the hydrogel to eradicate bacteria by burst release strategy.

**Methods:**

The MECs was coated with MDP1 (MECs-MDP1), characterized, and the hydrogel-peptide interaction was evaluated by molecular docking. Antibacterial activities of MECs-MDP1 were evaluated against VRSA and MRSA bacteria and compared to MECs-vancomycin (MECs-vanco). Antibiofilm activity of MECs-MDP1 was studied by our novel ‘*in situ* biofilm inhibition zone (IBIZ)’ assay, and SEM. Biocompatibility with human dermal fibroblast cells (HDFs) was also evaluated.

**Results and Discussion:**

Molecular docking showed hydrogen bonds as the most interactions between MDP1 and MECs at a reasonable affinity. MECs-MDP1 eradicated the bacteria rapidly by burst release strategy whereas MECs-vanco failed to eradicate them at the same time intervals. Antibiofilm activity of MECs-MDP1 were also proved successfully. As a novel report, molecular docking analysis has demonstrated that MDP1 covers the structure of MECs and also binds to lysozyme with a reasonable affinity, which may explain the inhibition of lysozyme. MECs-MDP1 was also biocompatible with human dermal fibroblast skin cells, which indicates its safe future application. The antibacterial properties of a photocrosslinkable methacrylated chitosan-based hydrogel coated with MDP1 antimicrobial peptide were successfully proved against the most challenging antibiotic-resistant bacteria causing nosocomial wound infections; VRSA and MRSA. Molecular docking analysis revealed that MDP1 interacts with MECs mainly through hydrogen bonds with reasonable binding affinity. MECs-MDP1 hydrogels eradicated the planktonic state of bacteria by burst release of MDP1 in just a few hours whereas MECs-vanco failed to eradicate them. inhibition zone assay showed the anti-biofilm activity of the MECs-MDP1 hydrogel too. These findings emphasize that MECs-MDP1 hydrogel would be suggested as a biocompatible wound-dressing candidate with considerable and rapid antibacterial activities to prevent/eradicate VRSA/MRSA bacterial wound infections.

## 1 Introduction

Wound infections can lead to complications such as cellulitis, necrosis, sepsis, multiple organ failure, and even death (Leaper et al., 2015). Wound infections occur after various pathologies, such as second (Deng et al., 2021) and third-degree burns (Bevalian et al., 2021), diabetes (Wei et al., 2021), surgery (Sun et al., 2020), and bedsores (Raisi et al., 2020).

The abovementioned wounds are highly susceptible to colonization by pathogenic bacteria (Edwards and Harding, 2004; Kalan et al., 2016; Pashaei et al., 2019). The most common bacterial species that cause skin infections in humans is *Staphylococcus aureus*, one of the most common nosocomial and community-acquired pathogens. *S. aureus* can hinder wound healing due to the production of various enzymes and toxins (Percival et al., 2012; Li et al., 2019). It is also prevalent to find methicillin-resistant *S. aureus* (MRSA) in skin infections (Taylor et al., 1992; Cook, 1998; Cohen et al., 2007). Vancomycin has been widely used as promising medicine to treatment of MRSA-associated skin infections for many years. However, the emergence of vancomycin -resistant *S. aureus* (VRSA) (Hiramatsu et al., 1997; Chang et al., 2003; Cong et al., 2020) and even resistant strains to its alternative antibiotics, daptomycin and linezolid (Kawasuji et al., 2023), has led to treatment failure in hospitalized patients. Along with the planktonic state, biofilm-producing *S. aureus* isolates are life-threatening in wound infections (De la Fuente-Núñez et al., 2013; Jamal et al., 2018). Biofilms prevent wound healing through bacterial infections, inflammation, dysfunction of fibroblasts, and collateral damage to surrounding tissues (Metcalf and Bowler, 2013; Lindley et al., 2016). More than 60% of chronic and 6% of acute wounds are infected by biofilm-producing bacteria (Zhao et al., 2013).

Conventional wound dressings have been utilized extensively in the medical field to prevent infections (Ong et al., 2008; Jayakumar et al., 2011; Wound Management Guidelines, 2016) but there are many frequent reports regarding their failure to protect against bacterial infections (Sweeney et al., 2012; Lei et al., 2019; Shi et al., 2020). A new generation of wound dressings is needed as a result of this issue. Hydrogels are widely used as soft functional materials in wound dressing and healing. Compared with conventional dressings, they offer moist pads, allowing oxygen diffusion (Ahmed, 2015; Kamoun et al., 2017; Liu et al., 2018; Xu et al., 2020), providing conditions for epithelial migration and granulation growth, accelerating tissue regeneration and repair (Li et al., 2021; Elyasifar et al., 2023). Chitosan is used in different forms such as hydrogel, cryogel, film and nanoparticle for various applications including scaffolds for cell culture, biosensor, tissue engineering, encapsulation and drug delivery (Piras et al., 2015; Acet et al., 2020; Rachtanapun et al., 2021; Taokaew et al., 2023). As biodegradable, biocompatible, and non-toxic, chitosan accelerates wound healing by improving the formation of granulation tissue along with angiogenesis, increasing the deposition of collagen fibers and epithelial thickness, and also inducing the production of growth factors (Ueno et al., 2001; Shi et al., 2006; Elyasifar et al., 2023). Physical, chemical, and biological properties of chitosan can be enhanced by incorporating methacrylate into its main chain. This modification enables the development of water-soluble chitosan that can be *in situ* crosslinked through light (Amsden et al., 2007; Zhao et al., 2014; Li et al., 2015). Photocrosslinkable hydrogels have received considerable attention in recent years for their application in the healing of wounds due to their advantages including their ability to be formed *in situ* in a minimally invasive manner, to form complex shapes that adhere to tissue structures (Elisseeff et al., 1999), improved mechanical properties as compared to physical crosslinking, and less toxicity than chemical crosslinking (Maitra and Shukla, 2014; Xu et al., 2018). Photocrosslinking through visible light offers notable advantages such as cost-effectiveness, safety, and tissue penetration due to its longer wavelength than UV light (Nguyen and West, 2002; Annabi et al., 2017; Kushibiki et al., 2021).

In recent decades, various antimicrobial compounds have been utilized in different scaffold with the aim of bacteria killing, inhibiting colonization, and preventing biofilm formation (Zafalon et al., 2018; Acet et al., 2023; Copling et al., 2023). Antibiotics are among the antimicrobial agents widely used in wound dressings (Grolman et al., 2019; Tamahkar et al., 2020; Tucker et al., 2021) but the misuse and overuse of these factors has led to the significant development of antibiotic-resistant bacteria through different mechanisms(Subramani and Jayaprakashvel, 2019; Acet et al., 2021). It is estimated that the number of deaths from infections caused by antibiotic resistance will reach 10,000,000 per year by 2050, which represents more deaths than all types of cancer (O’neill, 2014; Salam et al., 2023). Also quaternary ammonium species and silver nanoparticles are among the most popular alternatives to antibiotics in the treatment of infections, which have effective antibacterial activity (Yari et al., 2012; Kang et al., 2016; Li et al., 2016), but their toxicity hindered their applicability (Debbasch et al., 2001; Perani et al., 2001; Lam et al., 2004; Du Toit and Page, 2009).

Nowadays, modern wound dressings act not only as a protective layer but also as a therapeutic and healing system. Antimicrobial peptide (AMPs) as a promising agent, can invade bacteria through membrane damage by pore formation and depolarization as a main mechanism (Memariani et al., 2018), resulting in bacterial eradication, particularly against antibiotic-resistant strains (Brogden, 2005; Piotrowska et al., 2017; Carratalá et al., 2020; Magana et al., 2020). Additionally, AMPs exhibit antibiofilm activity, which can treat biofilm-associated infections (Batoni et al., 2016; Shams Khozani et al., 2018; Zarghami et al., 2021).

The addition of various AMPs to hydrogel wound dressings has received much attention in recent years to combat the problem of skin infections caused by antibiotic-resistant bacteria (Annabi et al., 2017; Yang et al., 2020; Atefyekta et al., 2021; Pati et al., 2021). In spite of their advantages, the clinical use of these dressings has been limited by a variety of factors including peptide toxicity and instability. Also, the use of inappropriate drug delivery systems including incorporating the peptide inside the hydrogel and requiring a period of time for the degradation of the scaffold to release the peptide, covalent immobilization of peptide to the surface of the hydrogel and limiting its access to infectious agents, as well as using sustained release instead of initial burst release systems, have led to the inability of these dressings to completely eradicate resistant bacteria (Annabi et al., 2017; Yang et al., 2020; Atefyekta et al., 2021; Pati et al., 2021). Developing a wound dressing containing a biocompatible peptide that completely eradicates resistant bacteria along with ideal bioavailability can overcome these challenges.

Melittin-derived peptide (MDP1), a mutant version of melittin, has recently developed and demonstrated that its hemolytic activity and cytotoxicity decreased by 100% and 72.9% compared to melittin (Akbari et al., 2018). Furthermore, MDP1 exhibited four times increased stability compared to melittin. The therapeutic index of this novel antimicrobial peptide has been determined to be 252 times higher than that of melittin (Akbari et al., 2022). Implementation of locally delivered AMPs by wound dressing has advantages including direct effect on infected skin without risk of damages to non-target tissues, high drug concentration at the skin wound site, and avoiding risk of bacterial resistance (Wu et al., 2022).

**FIGURE 1:**
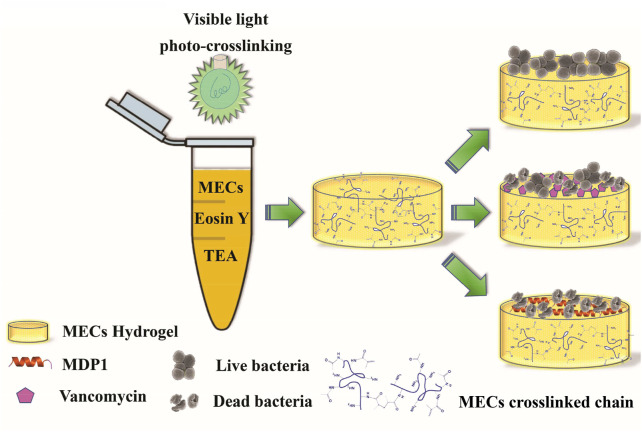
Schematic representation of the hydrogel synthesis and bacteria eradication.

Concerning the abovementioned limitations and clinical challenges of eradication of antibiotic-resistant life-threatening bacteria in wound infections, here, for the first time, we present this novel approach that the burst release of a fast-acting promising antimicrobial peptide at its biocompatible concentration can guarantee the complete eradication of antibiotic-resistant life-threatening bacteria. We hypothesized that the burst release of MDP1, as a novel fast-acting promising antimicrobial peptide, coated on photocrosslinkable methacrylated chitosan hydrogel with the favorable skin regenerative capacity may leads to rapid eradication of planktonic and biofilm states of VRSA and MRSA; the most challenging antibiotic-resistant bacteria found in chronic wound infections (Figure 1). Furthermore, we tried to decipher the mechanism of interaction between MECs and MDP1 by *in silico* molecular docking study. Finally, the biocompatibility of MECs-MDP1 as an antimicrobial wound dressing was investigated on human dermal fibroblast (HDF) cells.

## 2 Materials and methods

### 2.1 Materials, media, bacteria and cells

Chitosan (Cs, medium molecular weight (190,000-310,000 Da), degree of deacetylation ≥75%, high viscosity (200-800 cP), Sigma Aldrich, 448,877), methacrylic anhydride, Eosin Y, triethanolamine (TEA), N-hydroxysuccinimide (NHS), 1-(3-dimethylaminopropyl)-3-ethylcarbodiimide hydrochloride (EDC), Crystal violet, MTT, dialysis tubing (molecular weight cut off range of 12,000–14,000), and vancomycin were purchased from Sigma-Aldrich (Saint Louis, MO, United States). Glutaraldehyde was from DaeJung Chemical Co. (South Korea). Mueller-Hinton Broth (MHB), Mueller−Hinton Agar (MHA) and Tryptic soy broth (TSB) used for bacterial cultures were purchased from Merck (Darmstadt, Germany). Dulbecco’s Modified Eagle Medium (DMEM) and Fetal Bovine Serum (FBS) were from Gibco, Life Technologies (Grand Island, NY, United States). Smart BCA (Bicinchoninic acid assay) kit was purchased from Intron Biotechnology Co. (South Korea). *S. aureus* ATCC 29213 and multidrug-resistant (MDR) clinical isolates (VRSA and MRSA) were from our previous study (Bevalian et al., 2021). Human dermal fibroblast cell (ATCC PCS-201-012) was purchased from the national cell bank of Iran (NCBI, Pasteur Institute of Iran).

### 2.2 Peptide synthesis

A solid phase method and FMOC (9-fluorenyl-methoxycarbonyl) chemistry were utilized to synthesize MDP1 peptide (GIGAVLKVLTTGLPALIKRKRQQ) by an external facility (China Peptide Co., China). C-terminal amidation was performed on the peptide and reverse phase-high performance liquid chromatography (RP-HPLC) technique was applied to purify the peptide up to 97%. In addition, mass spectrometry was used to determine the molecular weight of the peptide. Concentration of the peptide was rechecked using Smart BCA assay kit according to manufacturer instructions.

### 2.3 Determination of MIC and MBC for MDP1

This assay was performed to select the efficient dose by which the examined bacteria is eradicated.

Minimal inhibitory concentration (MIC) and minimal bactericidal concentration (MBC) of MDP1 were determined against *S. aureus* ATCC 29213 along with clinical multidrug-resistant (MDR) bacterial isolates (VRSA and MRSA) (Zarghami et al., 2022). The examined bacteria were cultured in MHB medium at 37 °C for overnight and an initial suspension of the bacteria was prepared and adjusted to 0.5 McFarland turbidity (equivalent to 1.5 × 10^8^ CFU/mL at 625 nm) to achieve an optical density of 0.09. MDP1 was serially diluted in MHB in a 96-well microplate and a 100 µL of 1.5 × 10^5^ CFU/mL bacterial suspension was added to each well and incubated at 37 °C for 24 h. MIC and MBC were determined according to Clinical and Laboratory Standards Institute (CLSI), recommendations (Testing, 2019).

### 2.4 MDP1 toxicity assay

MTT assay was performed to evaluate the toxicity of MDP1 on HDF cells and also to select a non-toxic dose for further evaluations. Briefly, HDF cells were grown in DMEM medium enriched with 10% FBS and antibiotics (100 U/mL penicillin, 100 U/mL streptomycin) in an incubator at 37 °C with 5% CO2 and 95% humidity for 24 h. The cells were initially seeded at a density of 1 × 10^4^ cells/well and allowed to incubate for 24 h. The supernatants were removed and the cells were treated with 2-fold serially diluted concentrations of MDP1 in DMEM medium and incubated at 37 °C for an additional 24 h. Following this, MTT solution (0.5 mg/mL) was applied to each well for 4 h. After discarding the supernatants, a solution of 100 µL of isopropanol was added to each well and incubated at 37 °C with shaking for 20 min to dissolve the formazan salt. Finally, the absorbance was measured at 570 nm using a microplate spectrophotometer (Epoch-BioTek Co., Winooski, VT, United States). Untreated cells and cell free medium were used as positive and negative control, respectively. The percentage of cell viability was calculated based on Eq. (1):

$$\text{Viability }\%=\frac{{\text{OD}}_{\text{Test}}-{\text{OD}}_{\text{Negative}\;\text{Control}}}{{\text{OD}}_{\text{Positive}\;\text{control}}-{\text{OD}}_{\text{Negative}\;\text{Control}}}\times 100$$ (1)

### 2.5 Synthesis of methacrylated chitosan (MECs) and characterization by FTIR and ^1^H NMR

MECs was prepared following Samani et al.'s protocol (Samani et al., 2020). Briefly, chitosan was dissolved in distilled water and methacrylic acid at 60°C. pH was adjusted to 5.8-6 by adding NaOH. EDC, NHS, and methacrylic anhydride were added dropwise with a 2.5 M ratio of anhydride to amino groups. The solution was stirred overnight at room temperature. The MECs was dialyzed against deionized water for 3 days. In order to remove unreacted reagents, the dialysate was changed twice daily and freeze-dried for 24 h. The final product (a solid white cotton-like product) was stored at −20 °C.

To characterize MECs, FTIR (KBr method, Shimadzu, Japan) in the 4,000–400 cm^-1^ range was used to confirm the binding of methacryloyl moieties of methacrylic anhydride to chitosan.

The ^1^H NMR spectra were obtained using an Ultra Shield 500 MHz spectrometer (Bruker, Germany). Deuterated water (D_2_O) and D_2_O with HCL were used as the solvent for preparing MECs and Cs samples, respectively. The degree of deacetylation (D_D_ %) of native chitosan and synthesized MECs was calculated using Eq. (2) (ASTM F2260-18, 2018):

$$DD\%=\left[I_{H2D}/\left(I_{H2D}+\frac{1}{3}I_{HAC}\right)\right]\times 100$$ (2)

Where, I_HAc_ is the acetyl group’s integral at ca. 2 ppm, and I_H2D_ is the integral area of H_2_ proton on C_2_ carbon at ca. 2.8–3.0 ppm. The methacrylation degree (D_M_) of MECs calculated according to Eq. (3) (Samani et al., 2020):

$$D_{M}=D_{D\left(\text{Pure}\;\text{Cs}\right)}-D_{D\left(\text{MECs}\right)}$$ (3)

### 2.6 MECs-MDP1 hydrogel preparation

Lyophilized MECs concentration of 0.75% (w/v) was dissolved in distilled water containing 0.3% TEA (as a co-initiator) to prepare MECs hydrogels. Eosin Y (as a photoinitiator) and DTT (as a cross-linker) were added to the solution at final concentrations of 0.15 mM and 10 mM, respectively. Upon complete mixing, a definite volume of the solution was transferred into a mold with a diameter of 8 mm. To form MECs hydrogel, the solution was exposed to green visible light radiation at a wavelength of 525 nm for 4 min to crosslink methacrylate groups. Finally, MDP1 was dropcasted on the prepared hydrogel.

### 2.7 Experimental surface loading of MDP1 on MECs and *in silico* mechanism of their interaction by molecular docking analysis

MDP1 solution (2.03 and 4.06 µM) was dropcasted on the hydrogel surface (diameter of 8 mm) at 37 °C for 1, 3, 6, and 9 h. Then, each sample was washed three times with ultra-pure water. The washing solution was collected, and its peptide concentration (uncoated MDP1) was determined using Smart BCA assay kit according to manufacturer instructions. The amount of peptide loaded onto the hydrogel surface was calculated using Eq. (4) (Benedini et al., 2019):

$$\text{Loading}\%=C_{i}-C_{t}/C_{i}\times 100$$ (4)

Where C_i_ represents the initial peptide concentration and C_t_ represents the peptide concentration in washing solution in the abovementioned time points (t).

To study *in silico* mechanism of MDP1-MECs interaction by molecular docking, isomeric SMILES form of the chitosan (Two-Dimensional structure; 2D) was downloaded from pubchem (CID 71853, 9-mer glucosamine). Based on ^1^HNMR results, it was virtually deacetylated at a distinct percent and then methacrylated to generate MECs; both using ChemDraw suite (ver. 21.0.0.28). The obtained structure was dimerized by ChemDraw in order to simulate inter-atomic photocrosslinking of methacrylated groups between the MECs. Finally, the free energy of structure was minimized followed by prediction of its three-dimensional (3D) structure using Chem3D suite (ver. 21.0.0.28). Additionally, the 3D structure of MDP1 was predicted using the I-TASSER server (http://zhang lab.ccmb.med.umich.edu/I-TASSER/). The MECs and MDP1 were considered as receptor and ligand, respectively. All the non-polar hydrogen atoms were deleted by AutoDock Tools (ADT, ver 1.5.7) (Morris et al., 2009) and the polar hydrogens were then added. Docking assays were performed in triplicate by autodock vina software (Trott and Olson, 2010) and data presented as mean ± standard deviation. Finally, discovery studio was used to analyze the interaction between MDP1 and MECs. Interacting groups/atoms in MECs and amino acid residues of MDP1, the types of bonds and their distances were manually obtained by using discovery studio.

### 2.8 Characterization of MECs-MDP1 by ATR-FTIR and SEM

Coating of MDP1 on hydrogel surface was confirmed by ATR-FTIR analysis. This assay determined the newly formed functional groups in MECs-MDP1 in comparison to MECs. The IR spectra of the MECs-MDP1 hydrogel were recorded at 400-4,000 cm^-1^ by a FTIR instrument (Thermo Nicolet Avatar 360, United States).

Scanning Electron Microscopy (SEM) was used to assess the morphology of synthesized hydrogels (MECs and MECs-MDP1) using a SEM instrument (AIS2100C-SERON Technology, South Korea). To perform this analysis, the hydrogel samples were fixed with 2.5% glutaraldehyde and freeze-dried to ensure complete drying. The samples were coated with nanogold particles prior to visualization.

### 2.9 *In vitro* release of MDP1

In order to study of the *in vitro* release of MDP1 from the MECs-MDP1 hydrogel, PBS (100 µL) was added on the surface of samples at pH 5.5 and 7.4. The pH values of 5.5 and 7.4 were selected to mimic the wound condition. The samples were incubated at 37 °C for 3 days. At the time points of 1, 2, 3, 6, 12, 24, 48, and 72 h, a volume of 25 µL was collected from the samples and replaced with the same amount of PBS. Finally, the MDP1 concentration at each time points were measured using the BCA kit according to the manufacturer instructions and cumulative MDP1 release was calculated.

### 2.10 Biodegradation

The *in vitro* biodegradation of MECs-MDP1 hydrogels was performed in lysozyme-containing PBS solution (150 µL from 0.4 mg/mL lysozyme/PBS 1×, pH 7.4) to simulate wound exudate (Ren et al., 2005). The lysozyme solution was located on upward surface of the hydrogel samples and incubated at 37 °C for the time intervals of 1, 3, 5, and 7 days. The incubated solutions were removed from the hydrogels and the hydrogel samples were freeze-dried (alpha 1–2 LD plus; Martin Christ Gefriertrocknungsanlagen GmbH, Osterode am Harz, Germany). Finally, their weights were measured and the degradation percentage (DP) was then calculated according to Eq. (5) (Céspedes-Valenzuela et al., 2021).

$$\text{DP }\left(\%\right)=\left[\left(W_{t1}-W_{\text{tn}}\right)/W_{t1}\right]\times 100$$ (5)

### 2.11 Evaluation of antibacterial and antibiofilm activities of MECs-MDP1

#### 2.11.1 Colony-forming units and eradication assay

MECs-MDP1 hydrogels (diameter of 8 mm) were placed in a 96-well plate, each of MRSA, VRSA, and *S. aureus* ATCC 29213 bacteria (1.5 × 10^4^ CFU, 100 µL) was added to the wells, and incubated at 37°C for 3, 6, and 24 h. The bacterial suspension was collected and sub-cultured on MHA plates at 37°C for 24 h and finally, the number of colonies were counted. MECs and MECs coated with vancomycin (MECs-v) were used as negative and positive control, respectively. Experiments were performed in triplicate in each group.

Following the CFU assay, to verify that the hydrogels eradicate bacteria, the hydrogel was washed three times with MHB culture medium. Using a vortex device, the hydrogels were homogenized completely in 200 µL of the culture medium. Each of supernatant samples were subcultured on MHA medium and incubated at 37 °C for 24 h. Finally, the number of colonies was counted (Bevalian et al., 2021).

#### 2.11.2 Inhibition zones assay

A 100 µL of 0.5 McFarland bacterial suspensions i.e., *S. aureus* ATCC 29213, MRSA and VRSA (equivalent to 1.5 × 10 ^7^ CFU/mL) were cultured on MHA plates. The MECs hydrogels (diameter of 8 mm) were coated by distinct doses of MDP1 which were previously determined in CFU assay, placed on the agar plates in the downward direction, and incubated at 37 °C for 24 h. The inhibition zone diameter was then measured (Gao et al., 2020). Blank hydrogel (MECs) was served as negative control.

#### 2.11.3 *In situ* biofilm inhibition zone (IBIZ) assay for MECs-MDP1 hydrogels

This assay was innovated for the first time to evaluate the inhibition of biofilm on agar culture medium *in situ*. MRSA and VRSA strains (1.5 × 10 ^7^ CFU/mL) were cultured in TSA medium containing 1% glucose. MECs-MDP1 (diameter of 8 mm) were then placed on the cultured medium and incubated at 37 °C for 24 h, and the inhibition zone diameter was measured. MECs hydrogel was used as a negative control. The agar medium was then stained with crystal violet (0.05%) for 5 min to confirm biofilm formation on the plate surface and to prove the absence of biofilm around MECs-MDP1 hydrogels. The plate surface was washed three times with distilled water to remove the color from areas where biofilm had not formed and the zone diameter was then measured.

#### 2.11.4 Morphological assay by SEM

The mechanism of MECs-MDP1 on planktonic and biofilm state of MRSA and VRSA bacteria was investigated using SEM. Briefly, 10 µL of 0.5 McFarland bacterial suspensions (10^6^ CFU) were located on a lamella which located on the bottom of a flat 24 well microplate. The MECs-MDP1 hydrogels were placed downward on the lamella corresponding to each of planktonic and biofilm cultures and incubated at 37 °C for 2 and 24 h, respectively. The hydrogels were removed and the lamellae were fixed by glutaraldehyde (2.5%) at 4 °C for 2 h (for biofilm assay, the sample post-fixed in 1.5% osmium tetroxide for 1 h). The samples were washed with distilled water, completely dehydrated with increasing amounts of ethanol (20%–100%), coated with gold nanoparticles, and then examined in a SEM instrument (AIS2100C-SERON Technology, South Korea). MECs was used as a negative control.

### 2.12 Biocompatibility assay

Biocompatibility of the hydrogels was evaluated using HDF cells. According to ISO 10993-12, suitable amounts of MECs and MECs-MDP1 hydrogel samples were put in FBS-free DMEM for 1 and 7 days and the hydrogel extracts were collected at each time point. The MDP1 dose which eradicates the examined bacteria and showed no toxicity in MTT assay will be selected for coating on MECs in this assay. HDF cells (1×10^4^ cells/well) were cultured in 10% FBS-supplemented DMEM in a 96-well plate for 24 h under common culture conditions (37°C, 95% humidity, and 5% CO2). Then, supernatants were replaced with 100 µL of hydrogel extract and the cells were incubated for 24 h. MTT assay was performed to calculate the cell viability as mentioned before. Untreated cells and cell free medium were used as positive and negative control group, respectively.

### 2.13 Statistical analysis

All data were collected in triplicates and expressed as mean ± standard deviation (SD). By using one -way ANOVA and unpaired sample t-test, the difference between treatment and control groups were analyzed, and the *p*-values less than 0.05 were considered significant.

## 3 Results

### 3.1 MIC and MBC for MDP1

**FIGURE 2:**
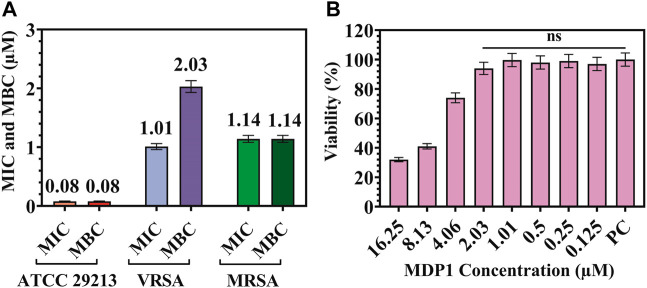
**(A)** MIC and MBC for MDP1 against *Staphylococcus aureus* ATCC 29213, VRSA, and MRSA strains. **(B)** MTT assay for MDP1. PC: Positive control. ns: non-significant.

This assay showed that MDP1 at the concentration of 0.08 ± 0.004, 1.14 ± 0.05, and 2.03 ± 0.09 µM eradicated the *S. aureus* ATCC 29213, MRSA, and VRSA, respectively. As shown in Figure 2A, MDP1 MICs and MBCs have been determined for each *S. aureus* strain.

### 3.2 MTT assay for MDP1

In order to evaluate the toxicity of different concentrations of MD on HDF cells, a MTT test was performed. The cells showed 94% ± 4.1% and 74% ± 3.3% viability up to the concentration of 2.03 and 4.06 µM of MDP1 for 24 h, respectively (Fig. 2B). In reference to 10993-12 standards (International Organization for Standardization, 2021), compounds are non-cytotoxic if their viabilities are ≥70% of the control group.

### 3.3 characterization of methacrylated chitosan by FTIR and ^1^H NMR

**FIGURE 3:**
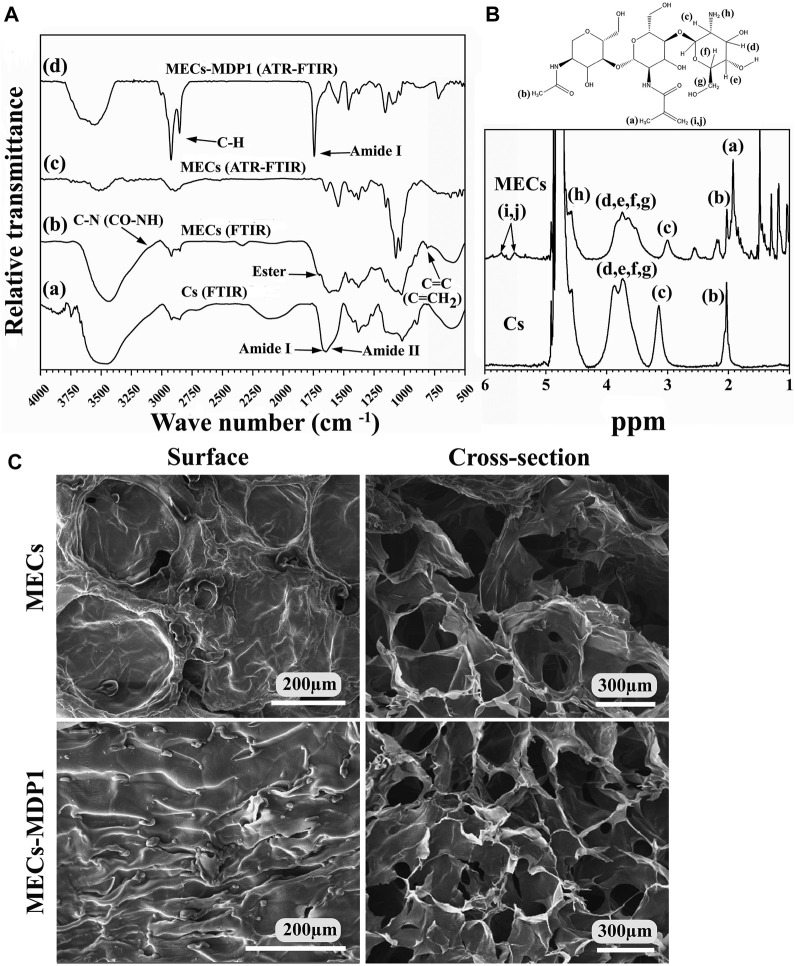
**(A)** Characterization of Cs, MECs, and MECs-MDP1. **(A, B)** FTIR spectra of Cs and MECs, respectively. (c, d) ATR-FTIR spectra of MECs and MECs-MDP1, respectively. **(B)** The ^1^HNMR spectra of Cs and MECs. **(C)** Scanning electron microscopy of the surface and cross-section of MECs and MECs-MDP1 hydrogels.

The identical peaks corresponding to chitosan (Fig. 3Aa) and methacrylated chitosan (Fig. 3Ab) were identified using FTIR. The peak of about 1,650 cm^-1^ is assigned to N-H bending of amide I (large amount of NH2 groups after chitin deacetylation and its conversion to chitosan) and amide II groups (acetyl groups of chitosan monomer) (Samani et al., 2020). The successful incorporation of methacrylate groups into chitosan was confirmed by the presence of signals at 1,620 and 845 cm^-1^ corresponding to C=C double bonds and also based on a decrease in the intensity of the amine type I stretching characteristic peak in the range of 1,600-1,639 cm^-1^ (Zanon et al., 2022; Elyasifar et al., 2023). Also, the increase in the peak intensity related to the C-N bond in CO-NH groups and the amide I group at 3,091 cm^-1^ (Zanon et al., 2022) is another confirmation of the successful methacrylation of chitosan. It should be noted that this group is present in chitosan due to the primary structure derived from chitin. It should intensify after methacrylation, as seen in MECs. The limited binding of MHA with chitosan’s hydroxyl groups through the formation of an ester bond was also shown at 1710 cm-1 (Zanon et al., 2022).

1H NMR spectroscopy analysis confirmed the chemical structure of MECs and determined the degree of chitosan methacrylation (Figure 3B). Both peaks at 1.98 ppm (marked as “b” in Figure 3B) and 3.4–4 ppm (marked as “c, d, e, f, g” in Figure 3B) represent methyl protons of N-acetylglucosamine (GlcNAc) and protons of glucosamine rings in Cs and MECs, respectively. Moreover, the peaks at 1.88 ppm in MECs (marked as “a” in Figure 3B) correspond to methyl protons (CH_3_) of methacrylate group (Samani et al., 2020). The peak related to the protons of the amine group (NH_2_, marked as “h” in Figure 2B) in Cs was located at about 4.5 ppm whose integration area in MECs decreased due to methacrylation through NH_2_ using EDC/NHS. Also, the peaks at 5.5–5.7 ppm (marked as “i”and “j” in Figure 2B) represent the protons of vinyl methylene (C=CH_2_) (Samani et al., 2020) and suggests that the methacrylate group successfully bonded to chitosan backbone through NH_2_ (Joshi et al., 2021). The deacetylation and methacrylation degree for Cs and MECs were about 70% and 40%, respectively.

### 3.4 Surface characterization of MECs-MDP1 hydrogel by ATR-FTIR and SEM

Figure 3 Ac and d, represent the ATR-FTIR spectra of MECs and MECs-MDP1, respectively. After coating of MECs surface with MDP1 by drop-casting, a new strong peak associated with type I amide band was appeared, which typically arises from the stretching vibrations of the C=O bond in the peptide backbone (Suo et al., 2021; Xiong et al., 2023). The exact location of the peak in this range can vary depending on specific interactions and peptide sequences. Also, the successful interaction of the MECs with MDP1 intensified the peak of C-H bonds in the range of 2,700-3,000 cm^-1^ which is due to the stretching vibrations of aliphatic C-H bonds in the infrared spectrum (Faraji et al., 2020), since hydrophobic amino acids (such as leucine, isoleucine, and valine) in MDP1 are rich in C-H bonds.

SEM determined the topography of freeze-dried MECs-MDP1 hydrogels. As seen in Figure 3C, the surfaces of MECs and MECs-MDP1 hydrogels had no porosity. MECs had a relatively smooth surface with some distortion which was well converted to a wrinkle surface by coating MECs with MDP1.

An interconnected porous network was found in the inner sections of MECs and MECs-MDP1 hydrogels. This structure can be considered an advantage for nutrients distribution. Moreover, due to the absence of MDP1 incorporation in the hydrogel, there was no significant difference in the size of the pores between MECs and MECs-MDP1.

### 3.5 Experimental peptide surface loading efficiency and *in silico* molecular docking analysis

**FIGURE 4:**
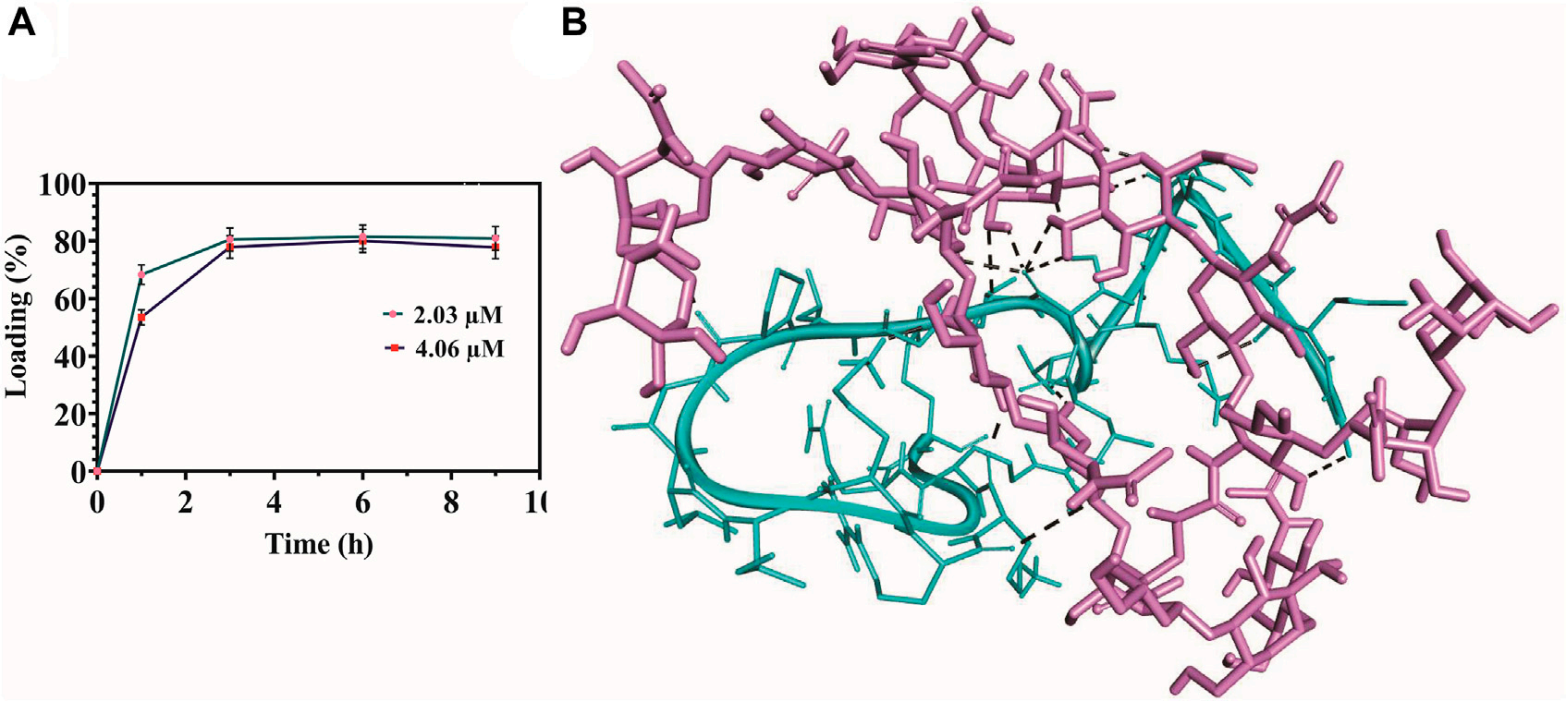
Peptide surface loading efficiency and molecular docking analysis. **(A)** Surface loading efficiency of MECs-MDP1. **(B)** Schematic representation of MDP1 molecular docking to MECs. The peptide’s binding affinity against MECs was −5.27 ± 1.4 kcal/mole. Light purple: MECs, Cyan: MDP1, Black spotted line: Bonding interactions.

The loading percent of MDP1 on the hydrogel surface was quantitatively determined by the BCA kit at the time intervals of 1, 3, 6, and 9 h. The amount of peptide loaded on the hydrogel surface reached its maximum after 3 hours; 80.5% ± 4.02% and 77.09% ± 3.85 for 2.03 µM and 4.06 µM, respectively (Figure 4A). Following this time point, loading efficiency entered the plateau state; 81.4% ± 4.07% and 80.02% ± 4 for 2.03 µM and 80.0% ± 4.1% and 77.8% ± 3.9 for 4.06 µM, after 6 and 9 h, respectively. On the basis of these results, the least time period in which the highest surface loading of MDP1 had been recorded was 3 h and this time period selected for all the following assays.

To perform molecular docking analysis, based on ^1^HNMR results, the 2D structure of Cs was deacetylated at the degree of 70% and then methacrylated at 40% using ChemDraw suite, sequentially. The peptide’s binding affinity against MECs was −5.27 ± 1.4 kcal/mole which indicates a moderate affinity (Wong et al., 2022). Interaction of MDP1 and MECs is demonstrated in Figure 4B. MECs interacted with MDP1 by hydrogen and electrostatic bonds in which, the most abundant bonds were hydrogen bonds. According to docking results, hydrogen bonds can form between the peptide backbone/side chain and chitosan functional groups, such as amine (-NH) or hydroxyl (OH) groups. The distance average for hydrogen bonds was 2.57 ± 0.48 Å, which indicates the proper interaction and binding affinity (Saunders et al., 2021).

Table 1 shows the MDP1-MECs interaction. Threonine, lysine, leucine, valine, alanine, proline, glycine, and arginine amino acid residues were responsible for MDP1 interaction with MECs. Fifteen interactions were documented in which hydroxyl groups of chitosan are the most abundant groups which interacted with MDP1; 53.33% of interactions. The other responsible groups/atoms were amine group (20%), glucose-amine ring’s carbon (13.33%), hydrogen from N-Acetyl (6.66%), and O-bridge (6.66%).

**TABLE 1:** MDP1-MECs interaction list.

Non-covalent interactions (Category)	Bond type (L-R)	Type	The numbers of bonds	Distance (Å)
Hydrogen Bond	O_L(R19)-_C_R(ring)_	Carbon Hydrogen Bond	3	3.44
	C_L(T11)_-O_R(OH)_			2.9
	O_L(P14)_-C_R(ring)_			3.55
	H_L(K18)_-O_R(OH)_	Conventional Hydrogen Bond	11	2.27
	O_L(K20)_-H_R(NH2)_			1.92
	O_L(V5)-_H_R(OH)_			2.19
	O_L(T10)_-H_R(NH2)_			2.67
	O_L(T10)_-H_R(NH2)_			2.85
	O_L(T10)_-H_R(OH)_			2.12
	O_L(L9)_-H_R(OH)_			2.17
	O_L(A4)_-H_R(N-acetyl)_			2.2
	O_L(T10)_-H_R(OH from_ _CH2OH)_			2.53
	O_L(I2)_-H_R(OH)_			2.81
	H_L(T10)_-O_R(O bridge)_			2.62
Electrostatic	N_L(G1)_-O_R(OH)_	-	1	3.35

R: Receptor (MECs), L: Ligand (MDP1). C-ring: Carbon in the glucosamine ring, N-acetyl: N-acetyl group in N-acetyl glucosamine. O-bridge: Oxygen flanked by glucosamine rings, R: arginine, T: threonine, P: prolin, K: lysine, L: leucine, A: alanine, I: isoleucine, G: glycine.

### 3.6 Releasing kinetics of MDP1

**FIGURE 5:**
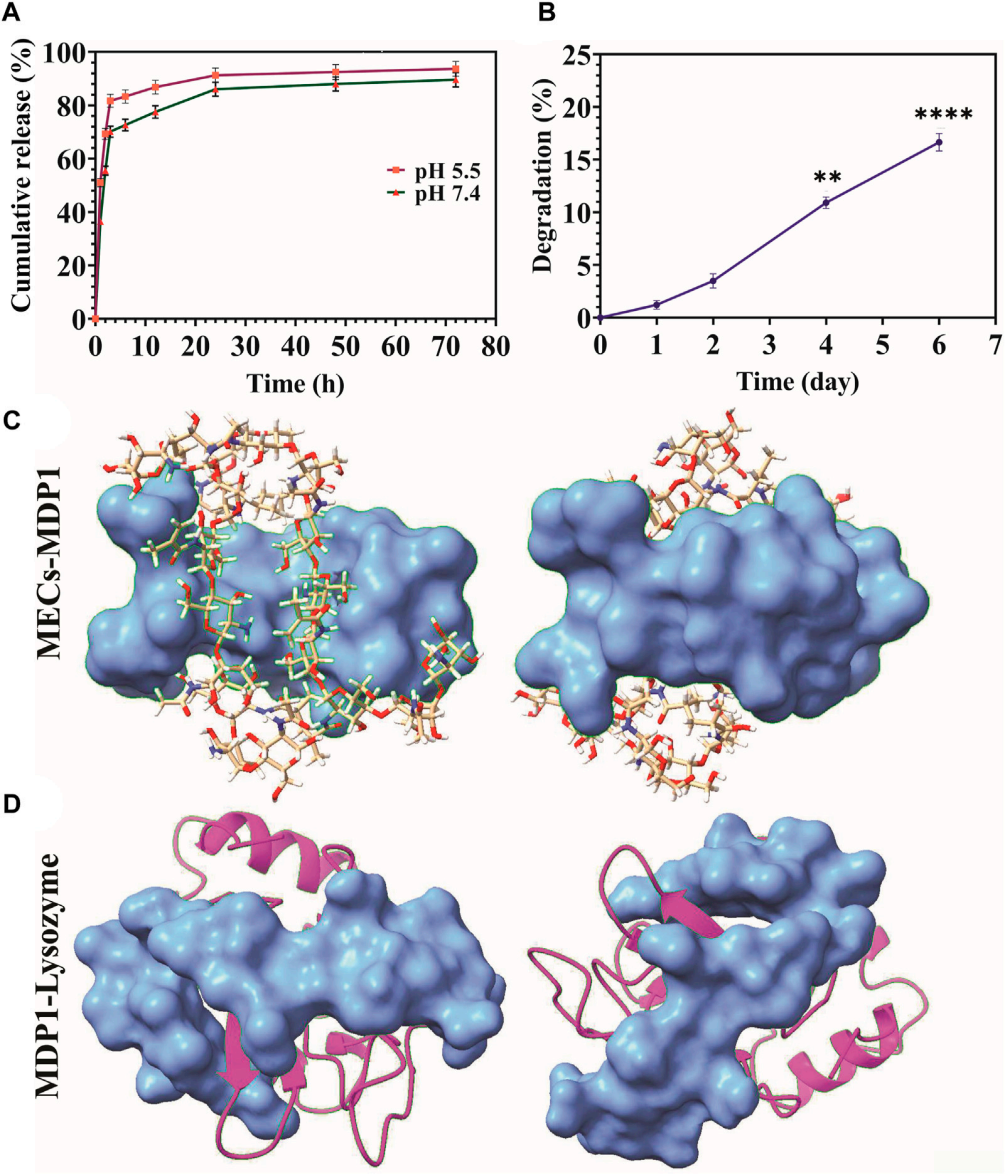
**(A)** Releasing kinetics of MDP1. **(B)** Biodegradation assay. A comparison of weight change has been made between all days and day zero. The data are presented as the mean ± SD (*p < 0.05, **p < 0.01, ***p < 0.001, n = 5 per group). **(C)** The coverage of MECs by MDP1. Ball-and-stick model: MECs, Blue surface model: MDP1 **(D)** Lysozyme-MDP1interaction. Magenta ribbon model: lysozyme, blue surface model: MDP1.

The release of MDP1 from MECs was performed in PBS at the pH of 5.5 and 7.4 at 37 °C for up to 72 h. The peptide (2.44 µM) at the pH of 5.5 and 7.4 had the burst release of 81.7% ± 2.45% and 70.12% ± 2.1 up to 3 h and then slowly reached to 93.7% ± 2.8% and 89% ± 2.69 up to 72 h, respectively (Figure 5A). Depending on the type of wound and the stage of the healing process, the pH level of the wound environment will vary significantly. The pH of the wound is generally acidic during the healing process, ranging from pH 5.0 to pH 6.5 (Tsukada, 1992). In chronic wounds, this range varies from 7.2 to 8.9 (Schneider et al., 2007; Bennison et al., 2017). The release of peptide was increased at pH 5.5 at all-time points.

### 3.7 Hydrogels biodegradation assay

Degradation of MECs-MDP1 in PBS + lysozyme was monitored for 6 days through weight loss measurement. The hydrogels experienced 16.65% ± 0.83% weight loss during 6 days of incubation in PBS + lysozyme (Figure 5B). Mass loss occurred at a greater rate from days two to six. According to molecular docking results, MDP1 can cover the structure of MECs and protect it from lysozyme interaction (Figure 5C). Also, MDP1 molecular docking to lysozyme showed a binding affinity of −5.4 ± 0.75, which indicates that MDP1 has a moderate inhibitory effect on lysozyme (Figure 5D).

### 3.8 *In vitro* evaluation of antibacterial activities of MECs-MDP1

To investigate the efficiency of MECs-MDP1 hydrogels, the *in vitro* antimicrobial activities were tested on VRSA and MRSA clinical isolates *S. aureus* ATCC 29213 was used as control.

#### 3.8.1 CFU and eradication assay

**FIGURE 6:**
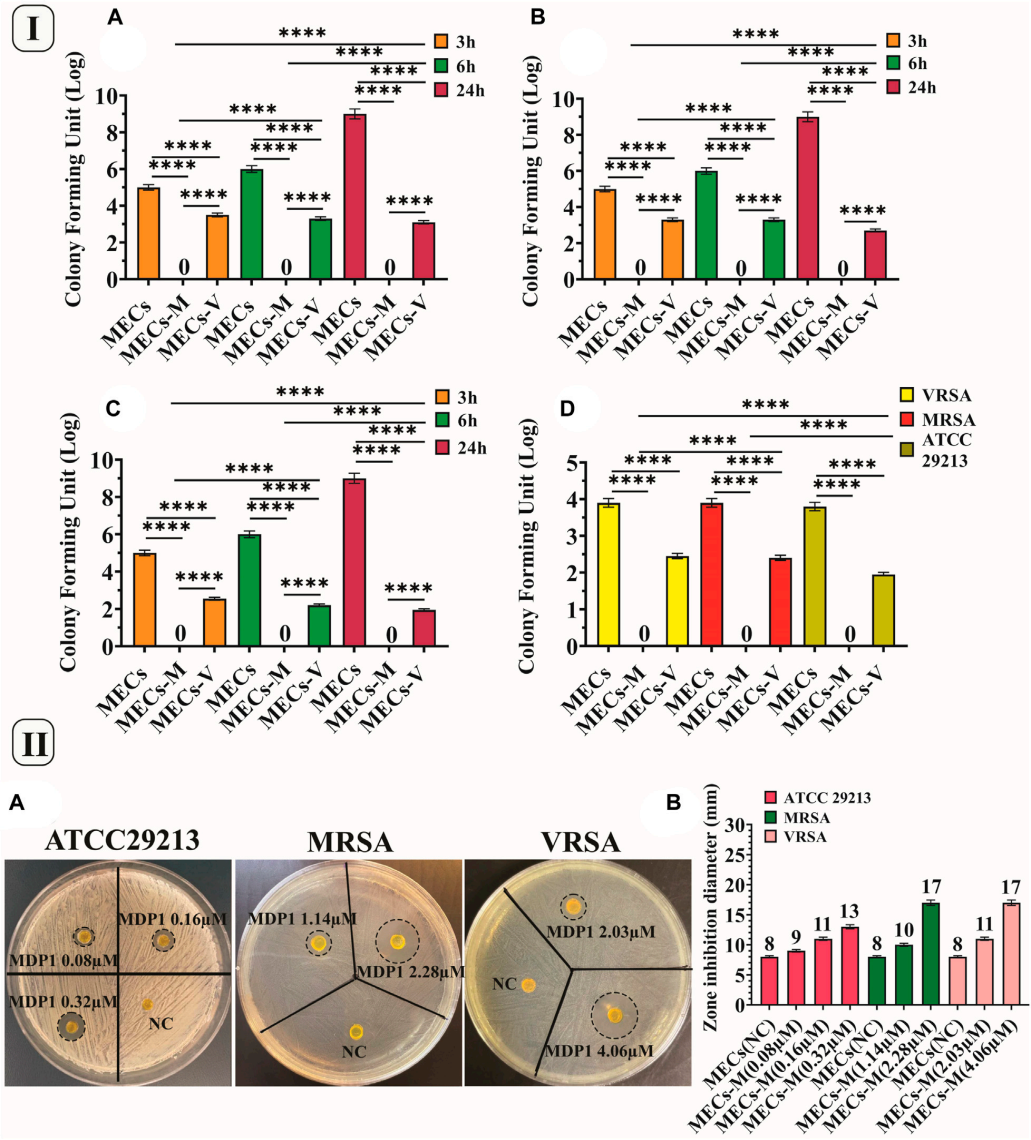
**(I)** CFU and eradication assay. CFU assay of the hydrogels against **(A)**VRSA, **(B)** MRSA, and **(C)**. *Staphylococcus aureus* ATCC 29213. **(D)** Eradication assay of the hydrogels against VRSA, MRSA and *Staphylococcus aureus* ATCC 29213, MECs-M. MECs-MDP1, MECs-V. MECs-vancomycin. Data is represented as mean ± SD (*p < 0.05, **p < 0.01, ***p < 0.001 and ****p < 0.0001, n = 3 per group). **(II)**. Investigating the zone inhibition formation by MECs-MDP1 for VRSA, MRSA and *Staphylococcus aureus* ATCC 29213 **(A)**. The zone diameter of MECs-MDP1 hydrogels **(B)**. NC: Negative control, MECs-M: MECs-MDP1.

The antibacterial activity of MECs-MDP1 was evaluated using the CFU assay against *S. aureus* ATCC 29213, MRSA, and VRSA at the concentration of 0.08, 1.14, and 2.03 µM, respectively. A remarkable finding was the complete killing of all three bacterial strains over 3 h by MECs-MDP1 (Figures 6IA–C) which indicates the fast-acting activity of MDP1. No colonies were also seen up to 24 h. However, MECs-vanco showed weak antimicrobial activity during 3 h in the same concentrations as MDP1 and finally failed to eradicate all examined strains over 24 h.

Following CFU assay, the hydrogels were homogenized in MHB medium to examine their eradication activities. It was observed that MECs-MDP1 eradicated 100% of all three strains (Figure 6ID). In contrast, MECs-vanco showed growing colonies. MECs-MDP1 has a significantly higher potential than MECs-vanco for eradicating the examined bacteria while comparing them at the same concentration and time intervals. MECs could not kill bacteria and uncountable bacterial colonies were seen. To confirm bacterial eradication, MECs-MDP1 was monitored for up to 48 h, and still, no colony growth was observed on the agar plates.

#### 3.8.2 Zone inhibition assay for planktonic state of bacteria

The TSA medium was used as a semi-solid surface in the zone inhibition assay. By using this assay, wound dressing hydrogels should be evaluated for their ability to prevent bacteria growth under and around the antimicrobial hydrogel (Gao et al., 2020).

According to Figures 6IIA, B, all MECs-MDP1 showed effective antimicrobial activity against all the three bacteria. The inhibition zone formed around MECs-MDP1 hydrogels proved that bacteria growth is inhibited around the hydrogels. Also the inhibition zone indicates MDP1 release from the MECs.

#### 3.8.3 *In situ* biofilm inhibition zone (IBIZ) assay for MECs-MDP1 hydrogels

**FIGURE 7:**
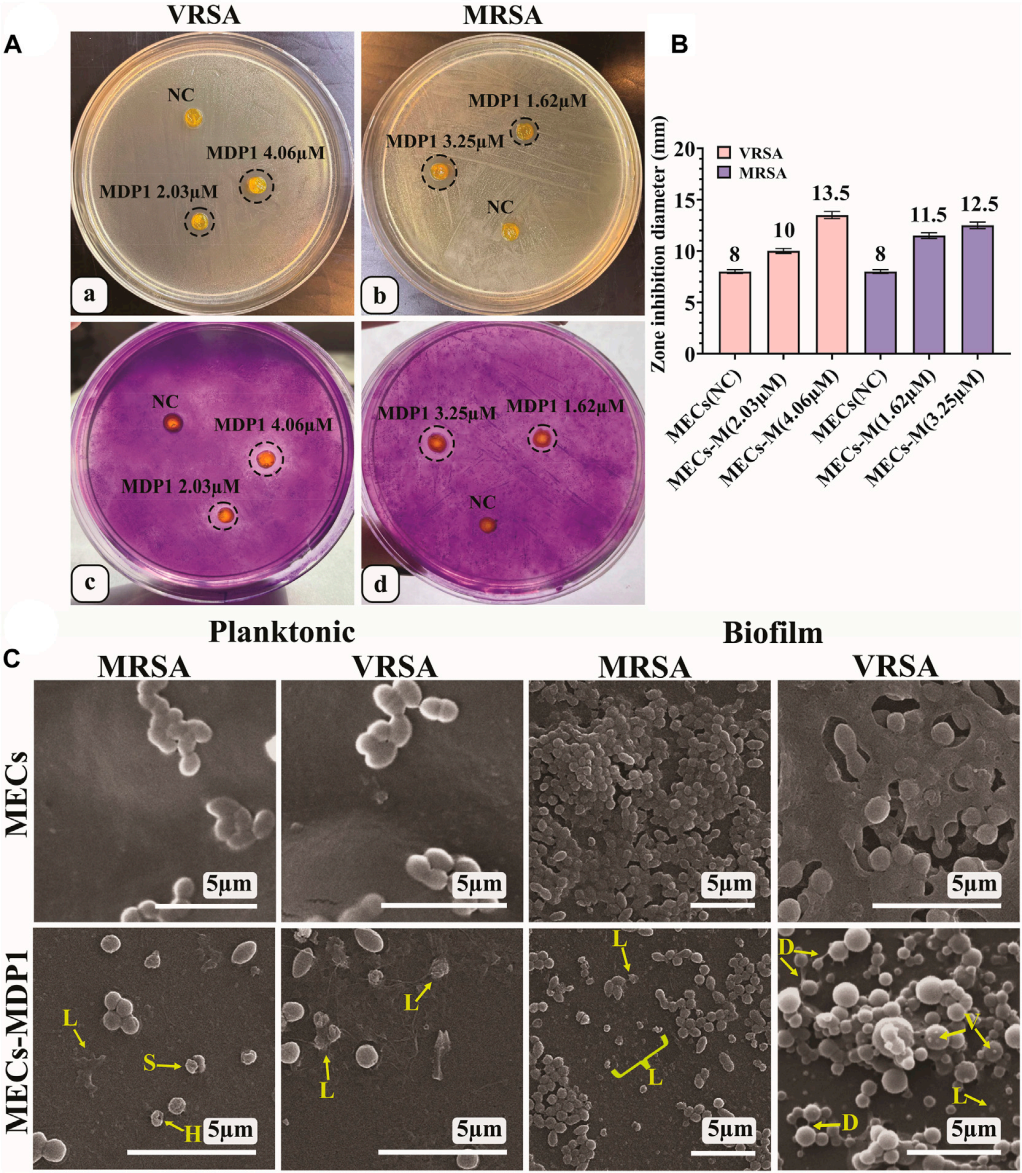
**(A)** IBIZ assay of MECs and MECs-MDP1 hydrogels for VRSA (a, c) and MRSA (b, d). **(B)** The zone diameter of MECs and MECs-MDP1 hydrogels. NC: Negative control, MECs-M: MECs-MDP1. **(C)** Investigation of the antibacterial and anti-biofilm formation of MECs and MECs-MDP1 by SEM. Morphological alterations were seen as vesiculation (V), bacterial lysis (L), bacterial detachment (D), and squeezing (S).

In the IBIZ assay, MECs-MDP1 hydrogels were used to inhibit biofilm formation by MRSA and VRSA bacteria using TSA medium containing 1% glucose. The zones of inhibition formed by MECs-MDP1 (‘4.06 µM and 2.03 µM for VRSA’ and ‘3.25 µM and 1.62 µM, for MRSA strain) are shown in Fig. 7Aa and b. All the MECs-MDP1 hydrogels inhibited biofilm formation of the examined bacteria. After measuring the zone inhibition (Figure 7B), the agar medium was stained with crystal violet to show the absence of biofilm formation around MECs-MDP1. As indicated in Fig. 7Ac and d), due to the presence of biofilm produced by VRSA and MRSA bacteria, a purple color was retained on the entire surface of the plate after three washes with distilled water except for the inhibited zone around the MECs-MDP1 hydrogel. According to the result, the plates have been completely covered with biofilm by the mentioned bacteria whereas the surrounding areas of the MECs-MDP1 have not been affected by bacterial growth or biofilm formation.

#### 3.8.4 Investigation of the antibacterial and anti-biofilm activity for MECs-MDP1 by SEM

This experiment was conducted to determine the inhibitory mechanism of MECs-MDP1 on the planktonic and biofilm state of MRSA and VRSA bacteria. The MECs-MDP1 altered the morphology of the planktonic state of MRSA and VRSA bacteria and destroyed them at the MDP1 concentration of 1.14 and 2.03 µM, respectively (Fig. 7C). Furthermore, no biofilm formation was observed in the MECs-MDP1 group at the concentration of 1.62 and 2.03 µM for MRSA and VRSA, respectively. Morphological alterations were seen as vesiculation, bacterial lysis, bacterial detachment, and squeezing.

### 3.9 Biocompatibility assay

**FIGURE 8:**
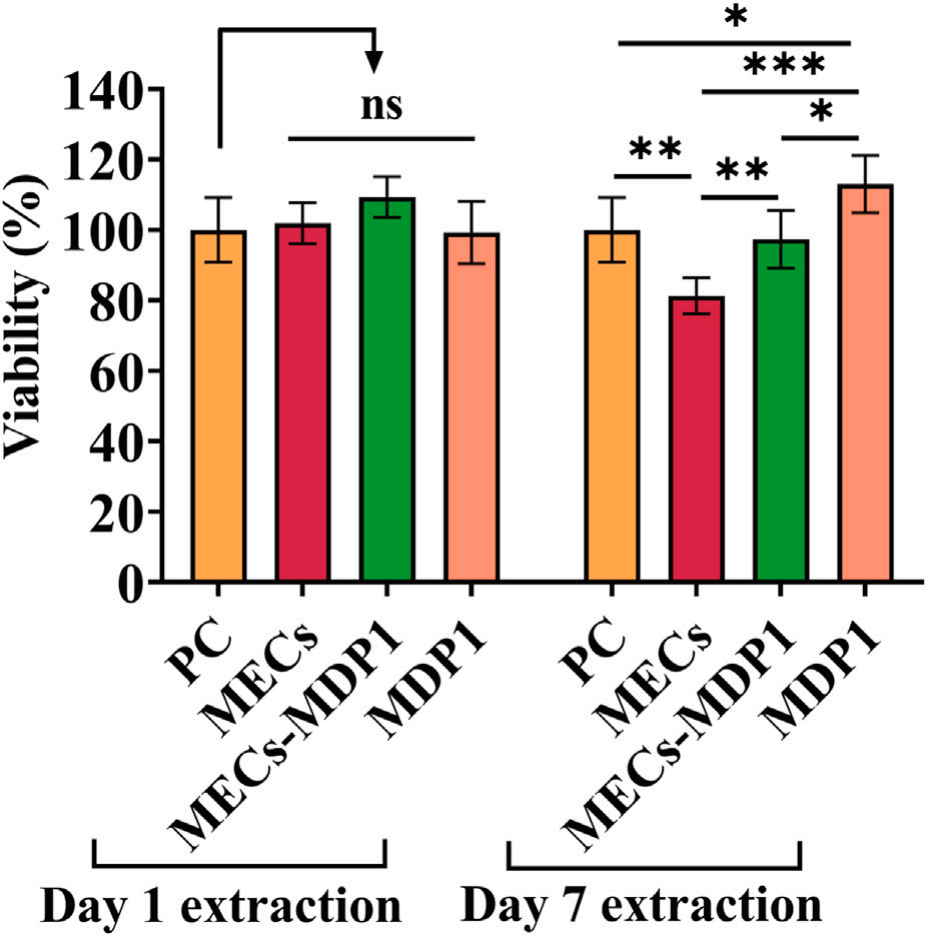
Cell viability of HDF cells incubated with 1- and 7-day extraction of MECs and MECs-MDP1. The medium was used as positive control (PC). The data are presented as the mean ± SD (*p < 0.05, **p < 0.01, ***p < 0.001, n = 5 per group).

The MTT assay was used to determine the viability of HDF cells and the safety of MECs-MDP1 wound dressings. The toxicity of MECs-MDP1 was evaluated at the MDP1 concentration of 2.03 µM. As shown in Figure 8, all 1- and 7-day extraction samples showed a high degree of biocompatibility exceeding regulatory biological toxicity standards. It was significantly higher in viability of the cells treated with 7-day extracts of MECs-MDP1 (97%) than in the cells treated with MECs (81%, *p* ≤ 0.006). Free MDP1 incubated in DMEM medium for 7 days induced more proliferation (113%) in comparison to 7-day extract of MECs-MDP1 (*p* ≤ 0.001).

## 4 Discussion

Referring to the frequent reports of antibiotic-resistant bacterial wound infections and its subsequent vital condition, a wound dressing harboring a promising antibiotic is of highly necessitated. In this study, we aimed at designing a photocrosslinkable methacrylated chitosan (MECs) hydrogel with the favorable skin regenerative capacity, coated by a novel fast-acting promising antimicrobial peptide, MDP1, to guarantee the complete and rapid eradication of planktonic and biofilm states of VRSA and MRSA; the most challenging antibiotic-resistant bacteria found in chronic wound infections by burst release strategy.

According to the activity and toxicity assays for MDP1, the concentrations of 2.03 and 1.14 µM are promising and safe for eradicating VRSA and MRSA without toxicity. Additionally, the MTT test demonstrated that MDP1 appears to be biocompatible with HDF cells up to the concentration of 4.06 µM.

Methacrylation of chitosan was confirmed by FTIR and ^1^H NMR. Following, MECs was coated by MDP1 which confirmed by ATR-FTIR. SEM images showed various phenomena such as surface wrinkles, shrinkage in the surface of MECs and MECs-MDP1 hydrogels. It might be due to the use of glutaraldehyde to fix the hydrogel samples. These phenomena are probably caused by re-crosslinking between aldehyde groups (-CHO) in glutaraldehyde and amine groups in chitosan, leading to the formation of Schiff base compounds (Beppu et al., 2007). It is important to note that the wound dressings are not subjected to extra preparation, such as fixation with glutaraldehyde and freeze-drying, before they are placed in clinical use. In comparison to MECs hydrogel, the abovementioned surface wrinkles were also seen on the surface of MECs-MDP1 hydrogel but to a greater degree. It is hypothesized that during peptide binding to the hydrogel matrix, it may cross-link the polymer chains together and cause such phenomenon.

Based on the molecular docking results, MDP1 showed a moderate affinity to MECs, with hydrogen bonds being the most prevalent bonds. Chitosan’s hydroxyl groups and amine groups are responsible for the majority of its interactions with MDP1, respectively. Non-covalent binding of MDP1 to MECs at the moderate affinity provide a condition for burst release of peptide to rapid eradication of bacteria. This could be an advantage to eradicate the colonized bacteria which also prevent the wound to be infected thus far (Zilberman and Elsner, 2008; Gimeno et al., 2015). However, according to the conditions of their use in clinical applications, the design of these wound dressings may require optimization or modification. The release of MDP1 in acidic condition would be due to the loss of interaction between MDP1 and MECs, which leads to faster peptide release. The isoelectric point (pI) of MDP1 is equal to 12.02. The pH values mentioned above are far lower than pI which leads to protonation of peptide (Novák and Havlíček, 2016). By increasing proton concentration, they compete with the previously-coated cationic peptide to detach them from the hydrogel and subsequent burst release.

According to biodegradation assay, it was demonstrated that MECs-MDP1 is only slightly degradable in lysozyme solution. Lysozyme is a glycoside hydrolase enzyme that is naturally present in various human tissues (Chen et al., 2005). It secretes by neutrophil in the infectious and non-infectious wounds (Tsai et al., 2021). It is also known that lysozyme degrades β(1-4) linkage between N-acetylglucosamine and glucosamine in MECs (Yin et al., 2021) whereas in this study MECs-MDP1 showed slightly degradation. Based on molecular docking studies, MDP1 provides a protecting barrier for MECs against lysozyme interactions. As well, a molecular docking study of MDP1 to lysozyme revealed a moderate binding affinity, which suggests that MDP1 has a reasonable inhibitory effect on lysozyme. In contrast to carbohydrate polymers that are highly susceptible to degradation by lysozymes, these characteristics of MDP1 could extend the expiration date of this type of wound dressing for future application.

The *in vitro* antimicrobial activities of the MECs-MDP1 hydrogels were evaluated using *S. aureus* ATCC 29213 and clinical isolates of VRSA, MRSA, the most challenging antibiotic-resistant bacteria causing nosocomial wound infections. The eradication of all three bacteria by MECs-MDP1 over 3 h represents an indication of the fast-acting activity of MDP1 which is consistent with the burst release of MDP1 from the MECs within 3 h. MECs-MDP1 has a significantly higher potential than MECs-vanco for eradicating the bacteria while comparing them at the same doses and time intervals. The remaining bacteria in the MEC-vanco group indicates that antibiotic resistance is a key factor in allowing infections to replicate and form biofilms. The lack of eradication of these strains by vancomycin makes patients vulnerable to SSTI infections, and also severe systemic infections such as septicemia and even death (Ladhani et al., 2021). Also, rapid eradication of bacteria by antimicrobial hydrogels with regenerating properties leads to accelerating the development of suitable conditions to expedite skin regeneration.

The antibacterial activity of MECs-MDP1 has also been confirmed by the formation of inhibition zones around MECs-MDP1 hydrogels, which indicates inhibition of the growth of all examined bacteria. Also, the inhibition zone confirms MDP1 release from the MECs.

The IBIZ assay indicated the inhibition of biofilm formation by MDP1. The result suggests that MECs-MDP1 has the potential to prevent biofilm formation by antibiotic resistant bacteria that cause serious infections of the wounds. In this assay the agar medium was stained with crystal violet to show biofilm formation on the surface of agar plates and the absence of biofilm formation around MECs-MDP1. Crystal violet dye typically carries a charge of +1 which can be bound to teichoic acids in Gram-positive bacteria by the peptidoglycan layer of the cell wall. Therefore, the areas of the agar surface without color indicate the absence of growth and biofilm formation. In the conventional methods to assess the anti-biofilm effect, it is necessary to place the hydrogel on the biofilm formed at the bottom of the well in a microplate. Thus, a part of the biofilm is removed from the bottom of the well due to the displacement of the hydrogel and caused an error in the measurement of anti-biofilm activity. Whereas, in IBIZ assay, the biofilm formation is examined *in situ*, as an inhibition zone surrounds the hydrogel without moving the gel and destroying the hydrogel integrity.

SEM showed a variety of morphological changes in bacteria such as vesiculation, bacterial lysis, bacterial detachment, and squeezing, which confirmed the anti-planktonic and anti-biofilm effect of MECs-MDP1. Similar phenomena have been observed for MDP1 and melittin in previous studies conducted by Akbari et al. and Bevalian et al. (Akbari et al., 2018; Bevalian et al., 2021).

As a final point, the MECs-MDP1 hydrogels showed no toxicity with the HDF cells which indicates its promising biocompatibility as a rapid antibacterial and skin regenerative wound dressing.

## 5 Conclusion

In summary, the antibacterial properties of a photocrosslinkable methacrylated chitosan-based hydrogel coated with MDP1 antimicrobial peptide were successfully proved against the most challenging antibiotic-resistant bacteria causing nosocomial wound infections; VRSA and MRSA. Molecular docking analysis revealed that MDP1 interacts with MECs mainly through hydrogen bonds with reasonable binding affinity. MECs-MDP1 hydrogels eradicated the planktonic state of bacteria by burst release of MDP1 in just a few hours whereas MECs-vanco failed to eradicate them. IBIZ assay showed the anti-biofilm activity of the MECs-MDP1 hydrogel too.

As a novel report, molecular docking analysis has demonstrated that MDP1 covers the structure of MECs and also binds to lysozyme with a reasonable affinity, which may explain the inhibition of lysozyme. MECs-MDP1 was also biocompatible with HDF skin cells, which indicates its safe future application.

Gathering all data together, MECs-MDP1 hydrogel is suggested as a biocompatible wound-dressing candidate to prevent/eradicate VRSA/MRSA bacteria rapidly which should be assessed in an organoid or *in vivo* model of wound infections.

## Data Availability

The raw data supporting the conclusion of this article will be made available by the authors, without undue reservation.

## Abbreviations

VRSA and MRSA, Vancomycin- and methicillin-resistant *S.taphylococcus aureus*; MECs, methacrylated chitosan; MDP1, melittin-derived peptide 1; MECs-vanco, MECs-vancomycin; IBIZ assay, *in situ* biofilm inhibition zone assay; HDFs, human dermal fibroblast cells; AMPs, Antimicrobial peptides; TEA, Triethanolamine; NHS, N-hydroxysuccinimide; EDC, 1-(3-dimethylaminopropyl)-3-ethylcarbodiimide hydrochloride; MHB, Mueller-Hinton Broth; MHA, Mueller−Hinton Agar; TSB, Tryptic soy broth.
