# Supplementary figures and images for: Rapid eradication of vancomycin and methicillin-resistant Staphylococcus aureus by MDP1 antimicrobial peptide coated on photocrosslinkable chitosan hydrogel: in vitro antibacterial and in silico molecular docking studies
Source: Front Bioeng Biotechnol. 2024 Apr 11;12:1385001. doi: 10.3389/fbioe.2024.1385001 (PMC11047131; doi:10.3389/fbioe.2024.1385001)

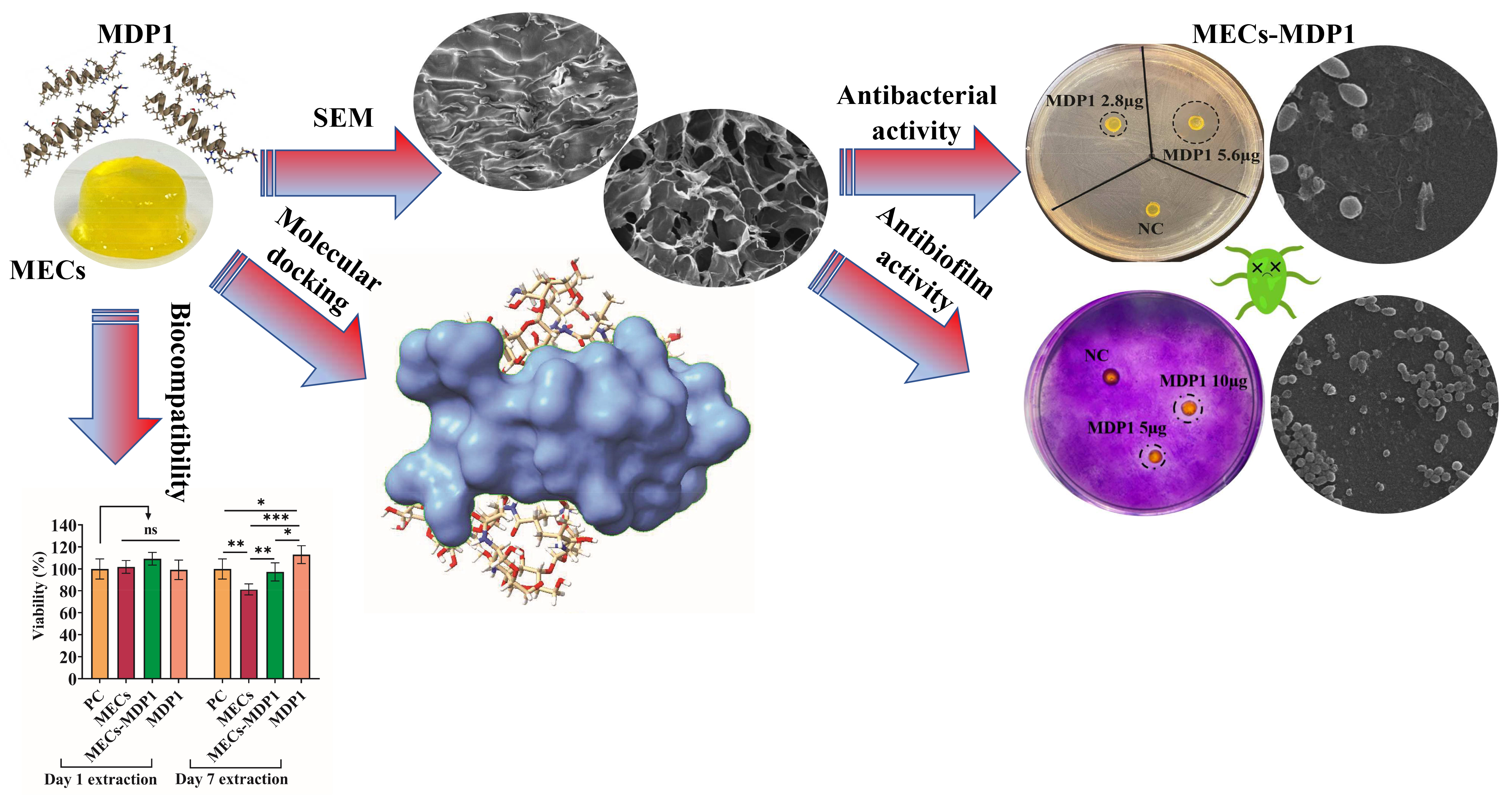

Supplement: Supplementary file 1 [file Image1.JPEG]
